# Assessing drug distribution in tissues expressing P-glycoprotein through physiologically based pharmacokinetic modeling: model structure and parameters determination

**DOI:** 10.1186/1742-4682-6-2

**Published:** 2009-01-15

**Authors:** Frédérique Fenneteau, Jacques Turgeon, Lucie Couture, Véronique Michaud, Jun Li, Fahima Nekka

**Affiliations:** 1 Faculté de Pharmacie, Université de Montréal, Montréal, Québec, Canada; 2 Charles River Laboratories Preclinical Services Montréal Inc., Montréal, Québec, Canada; 3 Centre de Recherche Mathématiques, Université de Montréal, Montréal, Québec, Canada; 4 Pharsight, Montréal, Québec, Canada

## Abstract

**Background:**

The expression and activity of P-glycoproteins due to genetic or environmental factors may have a significant impact on drug disposition, drug effectiveness or drug toxicity. Hence, characterization of drug disposition over a wide range of conditions of these membrane transporters activities is required to better characterize drug pharmacokinetics and pharmacodynamics. This work aims to improve our understanding of the impact of P-gp activity modulation on tissue distribution of P-gp substrate.

**Methods:**

A PBPK model was developed in order to examine activity and expression of P-gp transporters in mouse brain and heart. Drug distribution in these tissues was first represented by a well-stirred (WS) model and then refined by a mechanistic transport-based (MTB) model that includes P-gp mediated transport of the drug. To estimate transport-related parameters, we developed an original three-step procedure that allowed extrapolation of *in vitro *measurements of drug permeability to the *in vivo *situation. The model simulations were compared to a limited set of data in order to assess the model ability to reproduce the important information of drug distributions in the considered tissues.

**Results:**

This PBPK model brings insights into the mechanism of drug distribution in non eliminating tissues expressing P-gp. The MTB model accounts for the main transport mechanisms involved in drug distribution in heart and brain. It points out to the protective role of P-gp at the blood-brain barrier and represents thus a noticeable improvement over the WS model.

**Conclusion:**

Being built prior to *in vivo *data, this approach brings an interesting alternative to fitting procedures, and could be adapted to different drugs and transporters.

The physiological based model is novel and unique and brought effective information on drug transporters.

## Background

The most studied ATP binding cassette (ABC) membrane transporters is the P-glycoprotein (P-gp), which is a multidrug resistance (MDR) protein encoded by the ATP-binding cassette B1 (ABCB1) gene. The important role of P-gp in drug absorption and excretion in intestine, kidney and liver, has been revealed through reduction of absorption of orally administered drugs and promotion of urinary and biliary excretion [1,2]. Furthermore, P-gp transporters have a regulator function by limiting penetration of drugs in brain, heart, placenta, ovaries, and testes tissues. This has been shown *in vivo *on wild type (WT), mdr1a(-) and mdr1a/1b(-/-) knockout (KO) mice, which are mice lacking genes encoding for drug-transporting P-gp [3]. Indeed, higher levels of radioactivity were measured in various tissues of simple or double mutated mice compared to WT mice, after IV or oral administration of different P-gp substrates [3-8]. It has been demonstrated that modulation of the expression and/or activity of these transporters due to genetic or environmental factors may have a significant impact on drug disposition, drug effectiveness or drug toxicity [9-11]. Hence, characterization of drug disposition over a wide range of conditions of ABC membrane transporters activities is required to better characterize drug pharmacokinetics and pharmacodynamics.

Among pharmacokinetic modeling approaches, the physiologically based pharmacokinetic (PBPK) approach is now progressively used at various stages of drug discovery and development. PBPK models are developed to predict xenobiotic disposition throughout a mammalian body. By characterizing the kinetic processes of the drug, it is possible to predict its distribution inside tissues, organs and fluids of the body. The whole-body PBPK model involving tissues and organs connected via the vascular system mimics the anatomical structure of the mammal being studied. Generally, tissue distribution of drugs can be represented either by the perfusion rate limited (also called well-stirred) model, or the permeability rate limited model. The former assumes an instantaneous and homogenous drug distribution in tissues, whereas the latter represents the tissue as two or three well-stirred compartments which are separated by a capillary and/or cellular membrane where a permeability rate limited transfer occurs [12]. However, the membrane permeability may not be the only factor contributing towards limitation of drug distribution within a tissue. The influx or efflux activity of ABC transporters can be another important factor involved in drug distribution and should be considered as such in PBPK modeling.

In drug research and development, predicting drug disposition prior to *in vivo *studies is a major challenge [13]. Within this context, the hypothesis-driven strategy adopted here is to build a data-independent model that minimizes recourse to data fitting and exploits *in vitro *data information. Indeed, the spirit of PBPK modeling is deeply rooted in the independence of the model building on the output data representing the process to be described. It is based on the integration within a whole entity of drug specific characteristics with a structural mode which can be more or less detailed in terms of tissues and organs to be included. As relevant knowledge of the physiological, morphological, and physicochemical data becomes available, the possibility exists for efficient use of limited data in order to reasonably describe the pharmacokinetics of specific compounds under a variety of conditions [14]. With this in mind, the whole-body PBPK model developed herein aims to shed light, prior to *in vivo *experiments, on drug distribution in tissues expressing P-gp transporters. For this purpose, we adopt a step by step procedure which led us to the final PBPK model applied to mice, which accounts for the P-gp-mediated efflux transport in heart, and brain tissues. We first use the WS model to represent the drug distribution in each tissue. Then, to account for both passive and active transports, a mechanistic transport-based (MTB) model is developed for heart and brain. In order to estimate transport-related parameters all the while minimizing data fitting, we developed a method to extrapolate *in vitro *measurements of drug permeability of P-gp substrates through endothelial cells monolayers to the *in vivo *situation. This allowed the estimation of those parameters related to apparent passive and active transport of the drug through blood-tissue membrane of brain and heart.

To appreciate the reliability of the knowledge that the model provides in terms of elucidating the impact of the modulation of P-gp activity on drug distribution, we had access to WT and KO tissue concentrations of domperidone, an antiemetic drug associated with cardiac toxicity [15-17]. The choice of this drug model was motivated by previous *in vitro *results [18], which suggested that domperidone could be highly transported by P-gp. While this data set cannot be considered rich enough to validate the developed PBPK model, it can at least show that, the model simulations lie within realistic values by capturing points in the main strategic regions of the tissue concentration profiles, namely at the maximum concentration and the elimination phase.

## Methods

### Structure of the PBPK model

**Figure 1:**
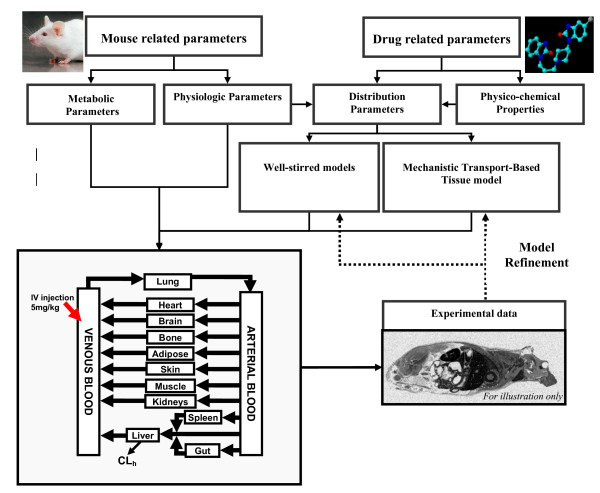
**Schematic representation of the procedures used to develop the whole body PBPK model applied to the mouse (30 g BW) following a 5 mg/kg IV injection of domperidone**.

The present investigation focuses on P-gp substrate distribution in heart and brain tissue where this transporter has a protective function. Our whole body PBPK model included these tissues as well as core tissues, organs and fluids, namely liver, arterial and venous blood, along with the adipose tissue because of its involvement in the disposition of lipophilic drugs. To make the model readily usable for subsequent updates and future experimental data, we also included bone, gut, lung, kidneys, muscle skin and spleen in the PBPK structure (Figure 1).

The PBPK model is mathematically formulated as a set of ordinary differential equations of mass balance that represents the time dependent variation of the drug concentration in each tissue. We systematically performed an overall mass balance of the whole-body PBPK model to assure that mass conservation laws are respected.

### Tissue-distribution models

The parameters used in the equations presented in this section refer to concentration (C), volume (V), blood flow to tissue (Q), tissue:plasma partition coefficient (P_tp_), blood:plasma ratio (BP), unbound fraction of drug (fu), clearance (CL), and permeability-surface area product (PSA). The subscripts refer to cardiac output (co), tissue (t), kidneys (k), spleen (sp), gut (g), plasma (p), liver (li), lung (lg), heart (ht), arterial blood (ab), venous blood (vb), blood in equilibrium with tissue (bl), venous blood living tissue (v, t), unbound fraction (u), bound fraction (b), intracellular water (iw), extracellular water (ew), neutral lipid (nl), neutral phospholipid (np), and microsomal binding (mic). Some subscripts refer to active transport processes, such as P-gp mediated transport (P-gp), as well as other transporters (OT) such as influx transporters (in, OT) and additional efflux transporters (out, OT).

#### Well-stirred model (WS)

At this first step of model development, the whole-body PBPK model is based on perfusion limited model of disposition. The uptake rate of the drug into tissues is limited by the flow rate to tissue rather than the diffusion rate across cell membranes [19]. In this case, the unbound concentration of drug in tissue is in equilibrium with the unbound drug in the outcoming blood. The application of a WS model requires the tissue-to-plasma partition coefficient (P_tp_) of each tissue included in the PBPK model as input parameters. By definition, these partition coefficients were calculated as:

(1) $${\text{P}}_{\text{tp,t}}=\frac{{\text{C}}_{\text{T}}}{{\text{C}}_{\text{p}}}=\frac{{\text{C}}_{\text{ut}}}{{\text{C}}_{\text{up}}}\frac{{\text{fu}}_{\text{p}}}{{\text{fu}}_{\text{t}}}={\text{fu}}_{\text{p}}\cdot {\text{Kp}}_{\text{u}}$$

where Kp_u _is the unbound tissue-to plasma partition coefficient [20] calculated from the tissue-composition-based approach developed by Rodgers et al. [20].

The hepatic elimination is determined from intrinsic clearance (CL_int_), such as

(2) $${\text{CL}}_{\text{int}}=\frac{{\text{V}}_{\text{max}\left(\text{P450}\right)}}{{\text{K}}_{\text{m(P450)}}}\times {\text{N}}_{\text{CYP450}}$$

where V_max(P450) _and K_m(P450) _are the Michaelis Menten parameters of drug biotransformation measured in mice hepatic pooled microsomes, and N_CYP450 _(nmol) is the amount of mice hepatic cytochrome P450.

The conventional description of hepatic extraction ratio (E_h_) corresponds to (CL_int _* fu_p_/fu_mic_)/(CL_int _* fu_p_/fu_mic _+ Q_h_) for a well-stirred liver model [21], where fu_mic _is the fraction of drug unbound to hepatic microsomes which can be estimated as follows for a basic drug [22]:

(3) **Fu_mic _= (C_mic_·10^0.56·LogP-1.41^+ 1)^-1^**

where C_mic _is the microsomal protein concentration (20 mg microsomal protein/mL herein), and LogP is the octanol:water partition coefficient of the drug.

The mass balance equations of the WS model applied to the tissues included in the PBPK model are [23]:

• non-eliminating tissues:

(4) $${\text{V}}_{\text{t}}\times \frac{{\text{dC}}_{\text{t}}}{\text{dt}}={\text{Q}}_{\text{t}}\times \left({\text{C}}_{\text{ab}}-{\text{C}}_{\text{v,t}}\right)$$

• eliminating tissues (liver)

(5) $$\begin{array}{c} {\text{V}}_{\text{li}}\times \frac{{\text{dC}}_{\text{li}}}{\text{dt}}=\left({\text{Q}}_{\text{li}}-{\text{Q}}_{\text{sp}}-{\text{Q}}_{\text{g}}\right)\times {\text{C}}_{\text{ab}}+{\text{Q}}_{\text{spl}}\times {\text{C}}_{\text{v,spl}}+{\text{Q}}_{\text{g}}\times {\text{C}}_{\text{v,g}}\\ -\frac{{\text{fu}}_{\text{p}}}{{\text{fu}}_{\text{mic}}}{\text{CL}}_{\text{int}}\cdot {\text{C}}_{\text{v,li}}-{\text{Q}}_{\text{li}}\times {\text{C}}_{\text{v,li}} \end{array}$$

where CL_int _and fu_mic _are estimated from equation 2 and 3 respectively.

• arterial blood

(6) $${\text{V}}_{\text{ab}}\times \frac{{\text{dC}}_{\text{ab}}}{\text{dt}}={\text{Q}}_{\text{co}}\times \left({\text{C}}_{\text{v,lg}}-{\text{C}}_{\text{ab}}\right)$$

• venous blood

(7) $${\text{V}}_{\text{vb}}\times \frac{{\text{dC}}_{\text{vb}}}{\text{dt}}=\sum_{\text{t}}\left({\text{Q}}_{\text{t}}\times {\text{C}}_{\text{v,t}}\right)-{\text{Q}}_{\text{co}}\times {\text{C}}_{\text{vb}}$$

• lung

(8) $${\text{V}}_{\text{lg}}\times \frac{{\text{dC}}_{\text{lg}}}{\text{dt}}={\text{Q}}_{\text{co}}\times \left({\text{C}}_{\text{vb}}-{\text{C}}_{\text{v,lg}}\right)$$

(9) $${\text{with C}}_{\text{v,x}}=\frac{{\text{C}}_{\text{x}}\times \text{BP}}{{\text{P}}_{\text{tp,x}}}\text{ where x stands for t, sp, li and lg}.$$

#### Mechanistic Transport-Based (MTB) models

We propose a transport-based tissue model to mechanistically investigate drug distribution in non-eliminating tissues expressing active transporters. This tissue model accounts for apparent passive diffusion and active transports of the drug at the blood-tissue membrane. Since only limited transport-related information is available within extra-and intra-cellular space of a tissue, it has been resumed by the transport occurring at the capillary membrane. This choice has the advantage to minimize the recourse to fitting procedures of transport-related parameters that would have been required in a three sub-compartmental tissue model. Thus, we assigned the term 'apparent' to the transport-related parameters and divided the tissue in two well-stirred compartments representing the vascular and extravascular tissues, separated by a capillary membrane where apparent diffusion and apparent active transports of the unbound drug occur. The fraction of drug unbound to tissue was calculated from the total tissue concentration C_T _estimated from the method developed by Rodgers and Rowland [20]. Indeed, C_T _can be expressed in terms of the unbound concentration in intracellular and extracellular water, and of the drug concentration bound to neutral lipid and phospholipids, such as [20]:

(10) **C_T _= C_u, iw_·f_iw _+ C_u, ew_·f_ew _+ C_b, nl_·f_nl _+ C_b, np_·f_np_**

The unbound drug fraction in tissues (fu_t_) was calculated by rearranging Equation 10, such as

(11) $${\text{fu}}_{\text{t}}=\frac{{\text{Cu}}_{\text{t}}}{{\text{C}}_{\text{T}}}=\frac{{\text{f}}_{\text{iw}}\cdot {\text{Cu}}_{\text{iw}}+{\text{f}}_{\text{ew}}\cdot {\text{Cu}}_{\text{ew}}}{{\text{C}}_{\text{T}}}$$

Remembering that Cu_ew _equals to the unbound concentration in plasma (Cu_p_), and Cu_iw _for a monoprotic base is given by [20]:

(12) $${\text{Cu}}_{\text{iw}}={\text{Cu}}_{\text{p}}\cdot \frac{\text{X}}{\text{Y}}$$

with

(13) **X = 1 + 10^(pKa-pHiw)^**

(14) **Y = 1 + 10^(pKa-pH)^**

Then, using equations 1, 11 and 12, fu_t _can be expressed as:

(15) $${\text{fu}}_{\text{t}}=\frac{{\text{f}}_{\text{iw}}\cdot \left(\frac{\text{X}}{\text{Y}}\right)+{\text{f}}_{\text{ew}}}{{\text{Kp}}_{\text{u}}}$$

where f_iw _is the fractional tissue volume of intracellular water and f_ew _fractional tissue volume of extracellular water. We used published tissue specific data [20], and assumed that the tissue composition in protein is the same among rodent (Table 1).

**Table 1:** Input physiological parameters used in PBPK model for IV injection of domperidone to a 30 g body weight mouse.

		**Tissue Composition (% of wet tissue weight) **[20]			**Physiological Data**			
**Tissues**	**Intra Cellular Water**	**Extra Cellular Water**	**Neutral Lipids**	**Phospholipids**	**Blood Flow Rate (% of Q_c_)^a^**	**Volume (% of BW)**	**Unbound Fraction to Tissue^b^**	**Partition Coefficient^c ^(Ptp)**
**Adipose^d^**	0.017	0.1350	0.853	0.002	0.07	0.0700	0.0079	1.7258
Bone + ROB*	0.346	0.1000	0.220	0.0005	0.218	0.0799	0.0327	2.0582
**Brain**	0.620	0.1620	0.031	0.05	0.031	0.0165	0.0463	2.5722
**Gut**	0.475	0.2820	0.032	0.015	0.141	0.0253	0.0166	6.2541
**Heart**	0.456	0.3200	0.017	0.014	0.066	0.0038	0.0212	4.8909
**Kidney**	0.483	0.2730	0.0148	0.0341	0.110	0.0135	0.0104	10.019
**Liver**	0.573	0.1610	0.0138	0.0303	0.161	0.042	0.0120	9.2366
**Lung**	0.446	0.3360	0.0218	0.0162	0.005	0.0073	0.0125	8.2560
**Muscle**	0.630	0.0790	0.0167	0.0273	0.159	0.384	0.0290	3.9387
**Skin**	0.291	0.3820	0.0239^d^	0.0180^d^	0.058	0.1653	0.0156	5.1585
**Spleen**	0.579	0.2070	0.012	0.0107	0.002^c^	0.0035	0.0184	6.3008
**Plasma**	---	---	0.096	0.0032	---	---	---	---
**Arterial blood**	---	---	---	---	---	0.0272^d^	---	---
**Venous blood**	---	---	---	---	---	0.0544^d^	---	---

^a^The mouse cardiac output value was estimated from the following allometric equation: Qc = 0.235 × BW^0.75^; ^b ^Calculated from equation 7.

^c ^Calculated from equation 1 using the method of Rodgers and Rowland[20]^d ^Rat value[23]; * ROB: rest of body

**Figure 2:**
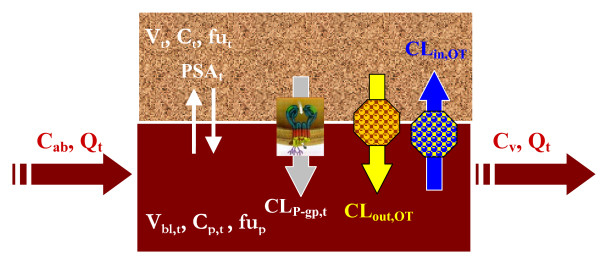
**Diagrams of the mechanistic transport-based tissue model that considers the passive transport of the drug, the P-gp mediated efflux transport, additional efflux transport and/or influx transport**.

The active transports include, but are not limited to, apparent P-gp mediated efflux of the unbound drug from tissue to blood. This general mechanistic transport-based model can also account for additional efflux (CL_out, OT_) and/or influx (CL_in, OT_) transporters. We first only consider the contribution of apparent passive diffusion and P-gp mediated transport in both tissues, setting thus to 0 the terms CL_in, OT _and CL_out, OT_. The transport-based tissue model can also be used to investigate the involvement of additional transporters by setting to non-zero values the parameters CL_in, OT _and CL_out, OT_. Compared to P-gp, there is limited knowledge for other transporters in terms of their activity and expression in mammalian tissues [24]. Hence, influx and/or efflux clearances of non P-gp transporters can be extracted from the best fit of tissue-concentration data. The general mass balance equations defining the mechanistic transport-based model applied to heart and brain tissues (Figure 2) are described below:

• Extravascular compartment (tissue)

(16) $$\begin{array}{c} {\text{V}}_{\text{t}}\times \frac{{\text{dC}}_{\text{t}}}{\text{dt}}={\text{PSA}}_{\text{t}}\times \left({\text{ fu}}_{\text{p}}\times {\text{C}}_{\text{p,t}}-{\text{fu}}_{\text{t}}\times {\text{C}}_{\text{t}}\right)-{\text{fu}}_{\text{t}}\times {\text{C}}_{\text{t}}\times \left({\text{CL}}_{\text{Pgp,t}}+{\text{CL}}_{\text{out,OT}}\right)\\ +{\text{ fu}}_{\text{p}}\times {\text{C}}_{\text{p,t}}\times {\text{CL}}_{\text{in,OT}} \end{array}$$

• Vascular compartment (blood)

(17) $$\begin{array}{c} {\text{V}}_{\text{bl,t}}\times \frac{{\text{dC}}_{\text{v, t}}}{\text{dt}}={\text{Q}}_{\text{t}}\times \left({\text{ C}}_{\text{ab}}-{\text{C}}_{\text{v,t}}\right)+{\text{PSA}}_{\text{t}}\times \left({\text{ fu}}_{\text{t}}\times {\text{C}}_{\text{t}}-{\text{fu}}_{\text{p}}\times {\text{C}}_{\text{p,t }}\right)\\ +{\text{fu}}_{\text{t}}\times {\text{C}}_{\text{t}}\times \left({\text{CL}}_{\text{Pgp,t}}+{\text{CL}}_{\text{out,OT}}\right)-{\text{fu}}_{\text{p}}\times {\text{C}}_{\text{p,t}}\times {\text{CL}}_{\text{in,OT}} \end{array}$$

### Mouse-related parameters

Mouse tissue composition, tissue volume, and blood-flow rate into tissue were extracted from the literature [25-27]; they are listed in Table 1.

The total amount of hepatic cytochrome P450 in mouse, N_CYP450_, was estimated by developing a log-log regression analysis that relates the total amount of N_CYP450 _of different mammalian species to their liver weight [28].

### Distribution-related parameters required for the MTB model

The volume of blood in equilibrium with brain and heart tissues (V_bl, t_) and the exchange surface area of the mouse blood-brain barrier were directly extracted from the literature [29-35]. Surface area (S_t_) per gram of cardiac tissue, only available for humans or quantifiable from human data [36,37], were applied to mice. As the estimation of permeability-surface area product (PSA_t_) and P-gp efflux (CL_P-gp, t_) clearance of a P-gp substrate through blood-tissue membrane is a crucial information, we have developed the following three-step procedure to estimate these parameters for mouse brain and heart tissue.

#### Step I: Estimation of in vitro diffusion and P-gp efflux rates of a P-gp substrate through Caco-2 monolayer

Assuming the drug is mainly transported by P-gp and used at a dose below the transporters saturation limit, then apical to basolateral apparent permeability (P_app, ab_) of drugs through Caco-2 monolayers results from the difference between apparent drug diffusion velocity (P_diff, *in-vitro*_) and apparent P-gp efflux rate (P_P-gp, *in-vitro*_). Basolateral to apical apparent permeability (P_app, ba_) is the result of the additive action of the drug diffusion velocity along with P-gp efflux transport. Assuming that P-gp efflux rate is independent of the direction of diffusion, the *in vitro *estimation of the parameters of apparent drug diffusion and apparent P-gp efflux rates (P_diff, *in-vitro*_and P_P-gp, *in-vitro*_) are calculated as follows:

(18) $${\text{P}}_{\text{diff, in-vitro}}=\frac{\text{1}}{\text{2}}\left({\text{ P}}_{\text{app, ba}}+{\text{P}}_{\text{app, ab}}\right)$$

(19) $${\text{P}}_{\text{Pgp, in-vitro}}=\frac{\text{1}}{\text{2}}\left({\text{ P}}_{\text{app, ba}}-{\text{P}}_{\text{app, ab}}\right)$$

where P_app, ba _and P_app, ab _values can be either directly measured through Caco-2 cells monolayers, or extracted from the literature.

#### Step II: In vitro-in vivo extrapolation of drug diffusion velocity and P-gp efflux rate parameters

We extrapolated *in vitro *P-gp efflux rate and diffusion velocity of P-gp substrates to the *in vivo *situation (Table 2), applying linear regressions procedures to data published by Collett et al. [38]. Some data presented in Table 2 are also extracted literature [39-45].

**Table 2:** Related parameters of the P-gp substrates used to establish linear regressions allowing the in *vitro-in vivo *extrapolation of diffusion and P-gp mediated efflux rates. Data were extracted from Collett and coworkers[38].

**Drug Name**	**MW**	**LogP**	**Papp_ab_^a, c ^cm/s**	**Papp_ba_^a, c ^cm/s**	**V_max(P-gp)_/K_m(P-gp)_^a, c ^cm/s**	**Pdiff_vitro _cm/s**	**Pdiff_vivo_^b ^cm/s**	**P_P-gp, vitro _cm/s**	**RAUC_corr_^b^**	**Ref**
Paclitaxel	854	3	2.1 × 10^-6^	8.61 × 10^-6^	2.1 × 10^-5^	5.36 × 10^-6^	3.04 × 10^-5^	3.26 × 10^-6^	3.26	[38,39]
Digoxin	789	2.2	1.1 × 10^-6^	7.15 × 10^-6^	1.3 × 10^-5^	4.13 × 10^-6^	3.08 × 10^-5^	3.03 × 10^-6^	1.03	[38,40]
Saquinavir	670	3.8	2.2 × 10^-6^	1.21 × 10^-5^	2.3 × 10^-5^	7.15 × 10^-6^	2.77 × 10^-5^	4.95 × 10^-6^	6.5	[38,41]
Topotecan	421	0.8	1 × 10^-6^	3.5 × 10^-6^	1.2 × 10^-5^	2.25 × 10^-6^	2.35 × 10^-5^	1.25 × 10^-6^	2*	[38,42]
Verapamil	454	4.7	1.5 × 10^-5^	1.5 × 10^-5^	0*	1.5 × 10^-5^	NA	0*	NA	[38,43]
Talinolol	363.5	2.9	1.5 × 10^-6^	1.5 × 10^-5^	1.5 × 10^-5^	6.0 × 10^-6^	NA	4.50 × 10^-6^	NA	[38,42,43,45]
Rifampicin	822	2.7	2.0 × 10^-6^	8.4 × 10^-6^	2.2 × 10^-5^	5.2 × 10^-6^	NA	3.20 × 10^-6^	NA	[38,42,43]
UK 224,671	544	1.8	3.0 × 10^-7^	8.4 × 10^-6^	9.1 × 10^-6^	3.2 × 10^-6^	9.43 × 10^-6^	2.88 × 10^-6^	32**	[38,42,45]

^a ^In Caco-2 experiments, the used drug concentration reported in Collett and coworkers[38] are 7.5 μM for saquinavir, 20 μM for verapamil and rifampicin, 30 μM for paclitaxel and digoxin, 40 μM for topotecan, talinolol and UK 224,671

^b ^In *in vivo *experiments, the dose administered to mice reported in Collett and coworkers [38] are 10 mg/kg of paclitaxel, 0.2 mg/kg of digoxin, 5 mg/kg of saquinavir and rifampicin, 2 mg/kg of UK 224,671, and 1 mg/kg of topotecan. Doses of verapamil and talinolol were not available.

^c ^pH 7.5 used in Caco-2 experiments[38]

* No secretion; ** assuming that RAUC reflects plasma ratio[38]

The authors measured P_app, ba _and P_app, ab _of some drugs through Caco-2 cells monolayer as well as P_app, ab _in the presence of a P-gp inhibitor (GF 120918). They determined the Michaelis-Menten kinetic parameters of active efflux transport, V_max(efflux) _and K_m(efflux)_, of these drugs. Moreover, they compared oral plasma area under the curve (AUC) of these compounds in WT and KO mice. In order to consider only the effect of P-gp on intestinal absorption of drugs, we corrected the ratio of drug AUC_oral _between species by removing the effect of P-gp involved in renal and biliary clearance on AUC_oral_. We first estimated the effect (E_IV-P-gp_) of the absence of P-gp on AUC_IV _measured after IV injection, such as:

(20) E_IV_-_P-gp _= (AUC_iv(KO) _- AUC_iv(WT)_)/AUC_iv(KO)_.

Then, the corrected ratio of oral AUC between both mice strains is calculated as follows:

(21) RAUC_oral, corr _= AUC_oral, KO, corr_/AUC_oral, WT_, = E_IV-Pgp _× AUC_oral, KO,_/AUC_oral, WT _

This ratio reflects the effect of P-gp mediated efflux in gut absorption:

(22) $${\text{R}}_{\text{AUCcorr}}=\frac{{\text{AUC}}_{\text{oral, KO, corr}}}{{\text{AUC}}_{\text{oral,WT}}}\approx \frac{{\text{Fabs}}_{\text{KO}}}{{\text{Fabs}}_{\text{WT}}}\approx \frac{{\text{P}}_{\text{diff, in-vivo}}}{{\text{P}}_{\text{diff, in-vivo}}-{\text{P}}_{\text{Pgp, in-vivo}}}$$

where F_abs _is the fraction of absorbed drug through the gastro-intestinal tract.

Then, we estimated *in vivo *diffusion velocity of these P-gp substrates through gut membrane from R_AUC, corr _value that we mechanistically approximated as follows:

(23) $${\text{P}}_{\text{diff, in-vivo}}\approx \frac{{\text{R}}_{\text{AUCcorr}}}{{\text{R}}_{\text{AUCcorr}}-\text{1}}\times {\text{P}}_{\text{Pgp, in-vivo}}≅\frac{{\text{R}}_{\text{AUCcorr}}}{{\text{R}}_{\text{AUCcorr}}-\text{1}}\times \frac{{\text{V}}_{\text{max }\left(\text{P-gp}\right)}}{{\text{K}}_{\text{m }\left(\text{P-gp}\right)\;}}$$

where P_P-gp, vivo _is approximated by the ratio V_max_(P-gp)/K_m_(P-gp).

We used the reported *in vitro *values of P_app, a-b _and P_app, b-a_, obtained in the presence and absence of P-gp inhibitor, to estimate P_diff, *in-vitro *_and P_P-gp, *in-vitro *_for each compound. Then, using S-Plus^®^, we assessed the correlations between *in vivo *V_max(P-gp)_/K_m(P-gp) _and P_P-gp, *in-vitro*_, and between P_diff, *in-vivo *_and P_diff, *in-vitro *_values of the drugs. These correlations are used to estimate apparent *in vivo *efflux rate of domperidone from P_P-gp, *in-vitro *_calculated in Step I.

As the tight junctions of the epithelium of the BBB contribute to the reduction of drug diffusion through this membrane, the diffusion velocity of the P-gp substrate under study through BBB was not estimated from measurement of apparent permeability through Caco-2 cells, but from *in vitro *measurement of its permeability through bovine brain capillary endothelial cells monolayer. This permeability value has been assigned a weight factor of 150, as suggested by Pardridge and coworkers [46] for in vitro permeability compared to in vivo permeability values measured in rats.

#### Step III: Calculation of the permeability-surface area product (PSAt) and P-gp-mediated efflux clearance (CL_P-gp, t_) of the P-gp substrate into mice brain and heart

The P-gp mediated efflux clearance has been found to be tissue-dependent [47]. Thus, P-gp expression levels in various tissues of WT mice [6] were used in our work to account for this tissue specificity. Since the Caco-2 cells line derives from human colon carcinoma and its characteristics are similar to intestinal epithelial cells, the intestinal tissue was chosen as the reference tissue for P-gp expression level. In each of the other mice tissues, the P-gp expression level has been estimated as a fraction of mice intestine P-gp expression (F_P-gp, t_,) and presented in Table 3[6]. We estimated CL_P-gp, t_, and PSA_t _both expressed in L/min:

**Table 3:** Additional physiological parameters required for the MTB tissue models applied to brain and heart.

**Tissue**	**V_bl_^a ^(mL/100 g tissue)**	**S_t_^b ^(dm^2^/g tissue)**	**F_P-gp, t_^c ^(-)**	**Cl_P-gp, t_^d ^(L/min)**	**PSA_t_^e ^(L/min)**	**Cl_out, OT_^f ^(L/min)**
**Brain**	2^g^	2^h^	0.42	3.71 × 10^-4^	3.56 × 10^-5^	2.8 × 10^-4^
**Heart**	20^i^	11.8^j^	0.26	2.61 × 10^-4^	1.2 × 10^-3^	---

^a ^Volume of blood in equilibrium with tissue

^b ^Exchange surface area

^c ^Relative fraction of mdr1a/1b mRNA expression in mice tissues compared to that in intestine, calculated from published data[6]. We calculated the ratio of multidrug resistance PCR product to that of β-actin in each organ and we related these ratios to that obtained in mice intestine tissue.

^d ^P-gp efflux clearance

^e ^Permeability-Surface area product

^f ^Parameter fitted to in vivo tissue concentrations

^g ^Intermediate value of published values: 1.6 uL/g brain [29]; 0.94 ug/g [30]; 3 ug/g [31]

^h ^Intermediate value of those published (1.50–2.40 dm^2^/g tissue) [32,33]

^i ^Rat value [34]. Same ratio was found in guinea pigs [35]

^j ^Human data applied to mice: Surface area of cardiac capillaries [36]

(24) $${\text{CL}}_{\text{Pgp, t}}=\frac{{\text{V}}_{\text{max }\left(\text{P-gp}\right)}}{{\text{K}}_{\text{m }\left(\text{P-gp}\right)\;}}\times {\text{S}}_{\text{t}}\times {\text{F}}_{\text{Pgp, t}}$$

(25) **PSA_t _= P_diff, *in-vivo *_× S_t_**

### Assessing drug distribution in tissues expressing P-gp

To investigate the ability of the developed PBPK model to assess the impact of P-gp activity modulation, we used tissue concentration of ^3^H-domperidone measured in adult male FVB WT and *mdr1a/1b *(-/-) KO mice after an IV injection at the target dose of 5 mg/kg. Blood, plasma, cerebral and cardiac tissue concentrations were available at 4 and 120 min post dose, while WT liver concentrations were available at 4, 7, 15, 30, 60 and 120 min post-dose. While the accessible data set in heart and brain tissues was limited in terms of the number of time points, it had the potential of asserting the quality of the model in those most strategic and informative regions of the lineshape, ie, near the peak concentration and at the elimination phase. We have also exploited a full data set available for WT liver to encompass the important aspect of hepatic disposition. The domperidone physicochemical characteristics required as input parameters to the model are extracted from literature [48-50]and presented in Table 4.

**Table 4:** Physico-chemical parameters of domperidone

**Physico-chemical parameters**	**Values**	**References**
Molecular weight	426	[48]
pKa	7.89	[48]
Octanol-Water partition coefficient (LogP)	3.35	EPIsuite [49]
Olive oil:water partition coefficient (LogP')	1.77^a^	[27]
Fraction unbound to plasma protein (fu_p_)	0.08	[50]
Blood:plasma ratio (BP)	0.92	[50]

^a ^Calculated from LogP' = (1.115 × LogP-1.35) [27]

## Results

### Estimation of metabolic parameters

**Figure 3:**
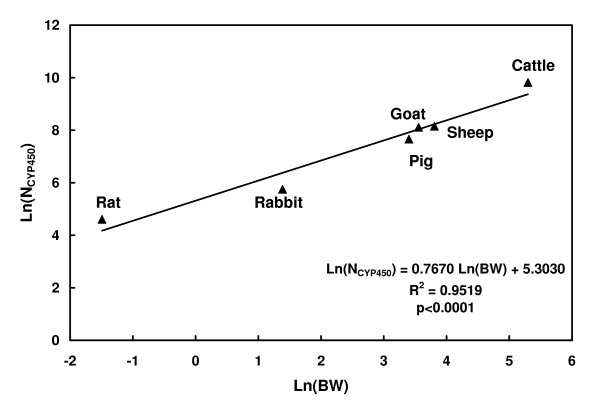
**Log-Log relationship between the amount of hepatic CYP450 and the body weight of various mammalian species**. Data from Craigmill et al., 2002 [28].

Since the drug was administered intravenously, the liver was considered as the only site of clearance by metabolism. We extrapolated N_CYP450 _to a value of 14 nmol for a 30 g BW mouse from the log-log regression calculated from published data [28] and presented in Figure 3. The kinetic parameters of domperidone biotransformation, K_m(P450) _and V_max(P450)_, were estimated to 130 μM and 4.6 nmol/nmolP450/min, respectively.

### Estimation of distribution parameters for WS and MTB models

**Figure 4:**
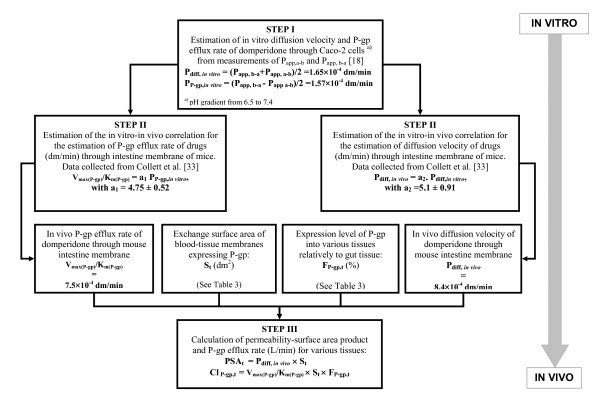
**Illustration of the three-step procedure developed to estimate *in vivo *apparent diffusion and P-gp efflux rates of domperidone through capillary membrane of the mouse brain and heart**.

The tissue-to-plasma partition coefficients of domperidone determined by the tissue-composition-based-approach [20] are listed in Table 1. Results of the three-step procedure developed above to estimate PSA_t _and CL_P-gp, t _rates of domperidone through blood-tissue membrane are presented in Figure 4. Positive linear correlations (V_max(P-gp)_/K_m(P-gp) _= 4.75 × P_P-gp, *in-vitro*_, R^2 ^= 0.92, no intercept, S-Plus^®^) were found between V_max(P-gp)_/K_m(P-gp) _and P_P-gp, *in-vitro *_as well as between P_diff, *in-vivo *_and P_diff, *in-vitro*_. (P_diff, *in-vivo *_= 5.1 × P_diff, *in-vitro*_, R^2 ^= 0.89, no intercept, S-Plus^®^). These correlations were used to estimate P_diff, *in-vivo *_and V_max(P-gp)_/K_m(P-gp) _of domperidone from P_P-gp, *in-vitro *_and P_diff, *in-vitro *_calculated in Step I. Finally, the third step gave rise to values of PSA_t_, and CL_P-gp, t _that we reported in Table 2 along with values of S_t _and F_P-gp, t_.

### WS Model

**Figure 5:**
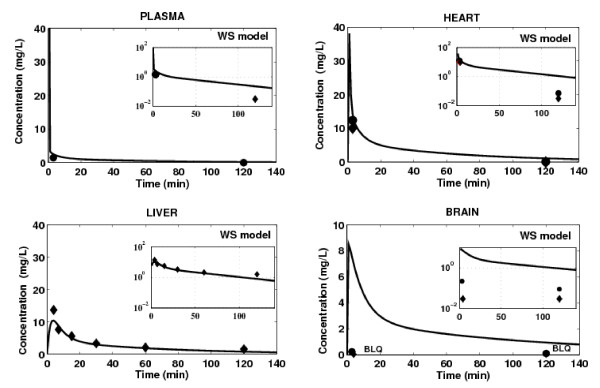
**Prediction of tissue concentration of domperidone using the WS model (black line) in any tissue/organ included in the PBPK model**. Tissue concentration measured in WT mice (black lozenge) and KO mice (black circle) after IV administration of 5 mg/kg of domperidone. BLQ = Below Limit of Quantification.

The concentration-time profiles of domperidone simulated in tissues using the WS model are presented in Figure 5. Only tissues for which experimental data were available are shown. The WS model successfully simulated the time-concentration profile of domperidone in hepatic tissue, indicating that the drug disposition in the main eliminating organ was adequately characterized. However, the WS model tends to overestimate domperidone concentrations in heart and brain tissues, which is likely to be related to a poor estimation of tissue-to-plasma partition coefficients for these tissues. The most important over-prediction of drug concentration is obtained in brain tissue. The predicted peak concentration in this tissue, regardless of the mice strain, was 8.5 mg/L, compared to a maximum measured concentration less than 0.03 mg/L and 0.22 mg/L, for WT mouse and KO mouse, respectively. As, by definition, this model is not suited to account for both active and passive transport mechanisms effect on drug distribution, a MTB model is applied to heart and brain tissues.

### MTB Models: Accounting only for P-gp Efflux Activity in Heart and Brain

**Figure 6:**
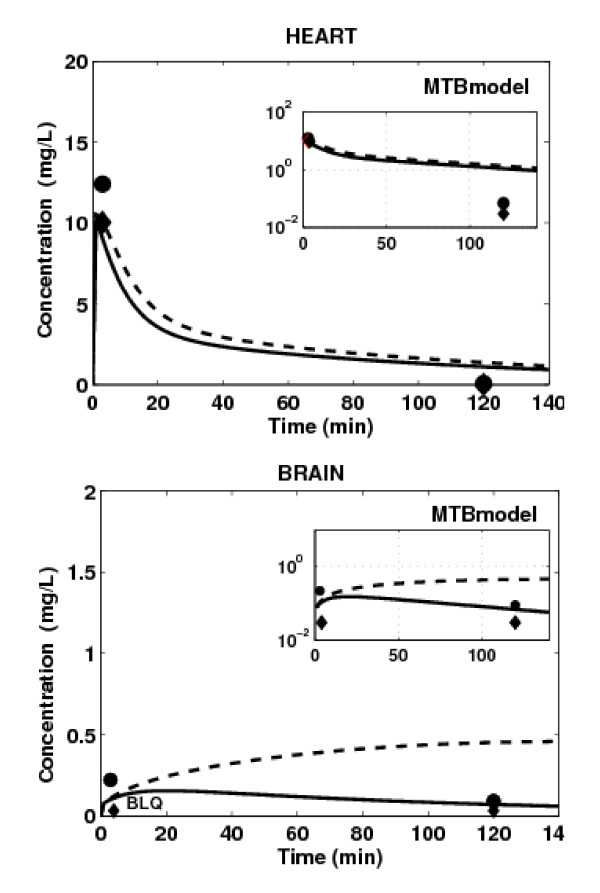
**Prediction of tissue concentration of domperidone in WT (black line) and KO (black dashed line) mice using the mechanistic transport based tissue model with passive and P-gp mediated efflux transports for heart and brain**. Tissue concentration measured in WT mice (black lozenge) and KO mice (black circle) after IV administration of 5 mg/kg of domperidone. BLQ = Below Limit of Quantification.

**Figure 7:**
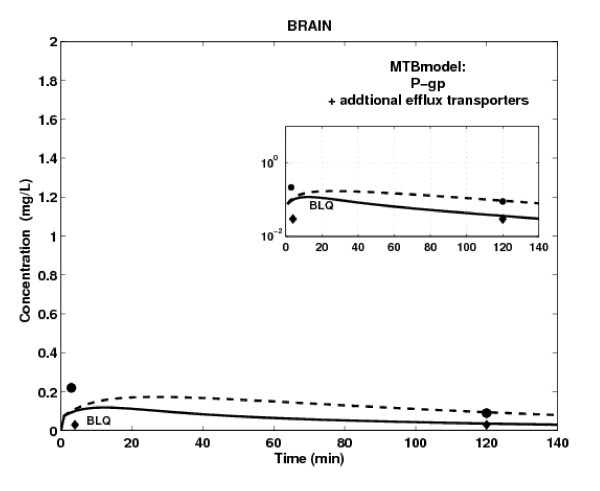
**Prediction of brain concentration of domperidone in WT (black line) and KO (black dashed line) mice using the MTB tissue model with passive transport, P-gp mediated efflux transport and additional efflux transport model for brain**. Tissue concentration measured in WT mice (black lozenge) and KO mice (black circle) after IV administration of 5 mg/kg of domperidone. BLQ = Below Limit of Quantification.

P-gp has a protective function by limiting drug accumulation into heart and brain tissues [1,2]. Therefore, we applied the MTB model to these tissues, and the WS model to all other tissues. The PBPK simulation results are illustrated in Figure 6. While the simulated effect of P-gp tends to be slightly lower than the observed one, the MTB model captures the peak concentration of domperidone for both mice strains in heart tissue. These results suggest that the apparent diffusion, rather than active transport, is the main transport mechanism of drug distribution in heart tissue. The MTB model significantly improves the WS model results in brain tissue, but it still tends to overestimate domperidone terminal concentration. In light of the above results, we were tempted to consider involvement of additional efflux membrane transporters in domperidone distribution in brain tissue (Figure 7). We derived its efflux clearance CL_out, O _by keeping diffusion and P-gp-mediated efflux parameters identical to those used for the brain MTB model while varying Cl_out, OT _parameter in order to fit simulated profiles to the available brain concentrations. In this case, the simulated concentration-time curves capture those terminal time points measured in brain tissue of both mice strains, but fail to reproduce the time-point concentration at 2 min post-dose. The trend of drug concentration profile in brain tissue simulated in the absence of P-gp activity but in the presence of additional efflux transporter is now in accordance with *in vivo *data (Figure 7, dashed line).

When compared to the WS model simulations, these results suggest that the apparent passive and active transport mechanisms are limiting processes of drug distribution in brain tissue.

The PBPK model that has been retained at the end of the modeling process comprises the MTB model for heart and brain tissues, and the WS model for all other tissues. When applied to heart tissue, the MTB model involves apparent passive diffusion and P-gp-mediated transports. For brain, the MTB model involves apparent passive diffusion, P-gp mediated transports and a potential additional efflux transport. However, this assumption should be further studied through a sensitivity analysis and additional *in vitro *and *in vivo *experiments.

## Discussion

The whole-body PBPK model developed herein aimed to shed light, prior to *in vivo *experiments, on drug distribution in tissues expressing ABC transporters, by including apparent active and passive transport processes. The model integrates the latest knowledge on the most studied ABC membrane transporters expressed in various tissues and organs. This is done by extrapolating *in vitro *drug permeability measurements across cells monolayers to *in vivo *conditions. This was performed with a three-step procedure proposed and developed herein, which allowed the estimation of the drug transport-related parameters without having recourse to data fitting. The proposed approach has to be used and interpreted with some caution in terms of the considered hypothesis and extrapolations. First, additional to P-gp, Caco-2 system can also express other transporters such as MRP and OATPs [51,52]. Hence, the *in vitro *estimated active transport rate may include the contribution of these additional transporters. However, it may be possible to isolate the effect of P-gp by adding a specific P-gp inhibitor, when performing Caco-2 experiments. Moreover, we have performed the in vitro-in vivo regression analysis of apparent diffusion and efflux transport by using a restricted data set [38]. Once additional information regarding Caco-2 essays and *in vivo *experiments using KO and WT mice becomes available for additional compounds, the quality and robustness of this analysis can be improved, reducing thus the uncertainty pertaining to the extrapolation procedure outside the range of permeability and drug efflux used for the correlation.

This study focused on the mechanisms of drug distribution in non-eliminating tissues expressing P-gp transporters, namely brain and heart. It was also prompted by the need to improve the ability of the PBPK approach to predict the impact of P-gp activity modulation on tissue distribution of P-gp substrates. Indeed, while the clinical importance of cardio-active agents in terms of efficacy and toxicity is well acknowledged, kinetics of drug transport into the myocardium has drawn little attention so far. Since many cardiovascular active compounds are subject to drug transport by ABC transporters, their expression in heart may strongly influence therapeutic or cardiotoxic effects [24]. However, the protective function of P-gp in heart tissue was not obvious from the present results.

Moreover, the multiplicity of drug transporters along with their complex nature at the BBB prevent a better understanding of the penetration mechanism of lipophilic compounds through this barrier [53]. Few physiologically based models have been developed to characterize drug distribution in brain tissues, mainly because of the complex anatomy of the central nervous system and the unavailability of physiological parameters [54,55]. Whereas the mechanisms involved in drug disposition into brain are not fully understood, some authors [56] have raised the potential benefit of using physiologically based compartment models to determine the rate of entry of drugs into and their distribution over the brain compartment. The proposed PBPK model pointed out to the protective function of P-gp against drug accumulation, which effect adds to the existing passive transport at the BBB.

So far, standard PBPK models have been generally composed of compartments that assume perfusion-rate limited (WS), permeability-rate limited, or sometimes, dispersion-rate limited models, the latter have not been discussed here. The WS principle was applied in this work as a first approximation model of drug distribution in each tissue included in our PBPK model. The main drawback of the WS model is its inability to capture the effect of transporters activity on P-gp substrate disposition. In such a case, its application can underpredict or overpredict drug concentration in target tissues [23]. This has been confirmed in the present study where the main deviation between the model predictions and the measured concentration of domperidone was observed in the brain tissue. This deviation can be attributed to the bias in the estimated brain-to-plasma partition coefficient value [26] since this coefficient does not account for active transport processes. Indeed, a significant overestimation of this parameter has already been noticed for another P-gp substrate, diazepam [23], and this bias translated into an overestimation of the brain concentration-time profile by at least a factor of three. However, this has neither been observed for ethoxybenzamide, a non-P-gp substrate, nor for propranolol [23], a P-gp substrate [57]. In the case of propranolol, P-gp was probably saturated [58,59] at the concentrations used [23], such that the diffusion process prevails on P-gp efflux transport. All this suggests that the WS model does not adequately describe disposition of P-gp substrate drugs in tissues where P-gp, when not saturated, have a significant protective function. Hence, it is natural to consider transport-based mechanisms as the next step in modeling domperidone distribution within the brain. These transport mechanisms can occur at the capillary or at the cellular membrane [12]. The cellular level of tissue subdivision can be used to investigate the impact of transporters activity modulation in drug distribution by including an influx/efflux clearance term at the cellular membrane [60]. However, this cellular subdivision asks for an increased amount of information which is rarely accessible without recurring to fitting procedures [12,60]. In the proposed MTB model, we divided non-eliminating tissues in two sub-compartments separated by the capillary membrane, where apparent passive diffusion and active transports occur, minimizing thus physiological information needed for passive and P-gp mediated active transports. This approach brings additional informative elements around the mechanisms involved in drug distribution within non eliminating tissues expressing P-gp.

## Conclusion

This paper was devoted to set up the fundamental mechanisms underlying distribution of drugs when active transporters are involved. The latest knowledge on P-gp transporters in heart and brain has been integrated. The proposed PBPK model has been defined for a mouse with average physiologic parameters, extrapolated within species and using *in vitro-in vivo *correlations. The next logical step in this process of model development will be to explore the behaviour of this PBPK model in terms of uncertainty and variability of its parameters. With the progress in acquiring quantitative knowledge on transporters, the procedure proposed in this work could be adapted for different drugs and transporters by taking into account their intrinsic characteristics.

## Abbreviations

The abbreviations of the parameters used herein refer to: (ABC transporters): ATP Binding Cassette Transporters; (BBB): blood-brain barrier; (BP): blood-plasma ratio; (BW in g): Body weight; (C in mg/L): drug concentration; (CL in L/min): clearance; (CYP450): cytochrome P450; (Eh): hepatic extraction coefficient; (F): fraction of expression level of a transporter in a tissue; (fu): unbound fraction of drug; (Km in μM): affinity constant; (KO): knockout-mice; (MTB): mechanistic transport-based model; (N_CYP450 _in nmol): amount of cytochrome P450; (P_app, ab _in dm/min): apical to basolateral apparent permeability through the Caco-2 monolayer; (P_app, ba _in dm/min): basolateral to apical apparent permeability through the Caco-2 monolayer; (PBPK): physiologically based pharmacokinetic; (P_diff, invitro _in dm/min): in vitro diffusion velocity of the drug through the Caco-2 monolayer; (P-gp): P-glycoprotein; (P_P-gp, invitro _in dm/min): in vitro P-gp efflux rate; (P_tp_): tissue-plasma partition coefficient; (PSA in L/min): permeability-surface area product; (Q in L/min): blood flow; (R_AUC, corr_): ratio of corrected plasma AUC measurements between WT and KO mice; (S_t _in dm^2^): exchange surface area separating vascular space from extravascular space; (V in L): volume; (V_max(P450) _in nmol/nmolP450/min): maximum velocity of CYP450 biotransformation; (V_max(P-gp) _in nmol/hr/cm^2^): maximum velocity of P-gp mediated efflux; (WS): well-stirred model; (WT): wild-type mice; The subscripts used refer to: (ab): arterial blood; (g): gut; (li): liver; (lg): lung; (ht): heart; (k): kidneys; (sp): spleen; venous (vb): blood; (p): plasma; (t): tissue; (bl, t): blood in equilibrium with tissue; (in, OT): other influx transporters; (out, OT): other efflux transporters; (int): intrinsic clearance; (mic): microsomes.

## Competing interests

The authors declare that they have no competing interests.

## Authors' contributions

FF has conducted the whole study including the results, outline, writing, and editing of the manuscript. The conception of this work has been conducted under the main supervision of FN who has been involved in the writing and revising this paper for its intellectual content. JT assured the co-supervision and access to experimental data collected on WT and KO mice, mainly provided by LC. VM contributed to measurement of Michaelis-Menten parameters of domperidone biotransformation in mice liver microsomes. JL contributed to the critic of the results and contents.
